# Water-Use Efficiency of Co-occurring Sky-Island Pine Species in the North American Great Basin

**DOI:** 10.3389/fpls.2021.787297

**Published:** 2021-12-03

**Authors:** Xinsheng Liu, Emanuele Ziaco, Franco Biondi

**Affiliations:** ^1^School of Geography and Tourism, Anhui Normal University, Wuhu, China; ^2^DendroLab, Department of Natural Resources and Environmental Science, University of Nevada, Reno, NV, United States; ^3^College of Tourism and Geography, Jiujiang University, Jiujiang, China; ^4^Department of Ecology and Genetics, Plant Ecology and Evolution, University of Uppsala, Uppsala, Sweden

**Keywords:** whole-tree transpiration, bristlecone pine, *Pinus longaeva*, limber pine, *Pinus flexilis*, subalpine forests, NevCAN

## Abstract

Water-use efficiency (WUE), weighing the balance between plant transpiration and growth, is a key characteristic of ecosystem functioning and a component of tree drought resistance. Seasonal dynamics of tree-level WUE and its connections with drought variability have not been previously explored in sky-island montane forests. We investigated whole-tree transpiration and stem growth of bristlecone (*Pinus longaeva*) and limber pine (*Pinus flexilis*) within a high-elevation stand in central-eastern Nevada, United States, using sub-hourly measurements over 5 years (2013–2017). A moderate drought was generally observed early in the growing season, whereas interannual variability of summer rains determined drought levels between years, i.e., reducing drought stress in 2013–2014 while enhancing it in 2015–2017. Transpiration and basal area increment (BAI) of both pines were coupled throughout June–July, resulting in a high but relatively constant early season WUE. In contrast, both pines showed high interannual plasticity in late-season WUE, with a predominant role of stem growth in driving WUE. Overall, bristlecone pine was characterized by a lower WUE compared to limber pine. Dry or wet episodes in the late growing season overrode species differences. Our results suggested thresholds of vapor pressure deficit and soil moisture that would lead to opposite responses of WUE to late-season dry or wet conditions. These findings provide novel insights and clarify potential mechanisms modulating tree-level WUE in sky-island ecosystems of semi-arid regions, thereby helping land managers to design appropriate science-based strategies and reduce uncertainties associated with the impact of future climatic changes.

## Introduction

Climatic variability can profoundly impact carbon and water exchanges between forests and the atmosphere (Frank et al., 2015). In arid and semi-arid ecosystems, both productivity and water cycling are co-limited by drought (Knowles et al., 2020). Tree species in such water-limited environments can adapt to climatic changes through a variety of physiological mechanisms, spanning from stomatal regulation to whole-tree remobilization of non-structural carbohydrates (Hartmann and Trumbore, 2016). When focusing on the trade-off between carbon gain and water loss, water-use efficiency (WUE), i.e., the amount of plant dry matter gained per unit water transpired, is a key characteristic of ecosystem functioning and a component of forest drought resistance (Law et al., 2002; Keenan et al., 2013; Ponce-Campos et al., 2013). Information on WUE is also needed for sustainable management strategies under changing climatic conditions in semiarid regions (Loehle et al., 2016; del Campo et al., 2017).

At the ecosystem level, complex WUE–drought relationships across broad ranges of biomes and environments have been linked to drought intensity, which alters the degree of coupling between gross primary productivity and evapotranspiration (Xu et al., 2019). For instance, tree ring-derived estimates of increasing WUE has been attributed to rising atmospheric CO_2_ concentration, but increased water stress could also induce a WUE increase in water-limited environments (Driscoll et al., 2020). At the same time, a reduction in WUE has been documented in drought-stressed trees (Linares and Camarero, 2012; López et al., 2021). Such discrepancies in WUE–drought relationships can also be the result of species-specific evolutionary histories, morphological traits, and/or physiological strategies (Yi et al., 2018), making studies on whole-tree WUE necessary to gain a comprehensive picture of tree physiology under a changing climate (Monson et al., 2010).

Tree-level WUE has been inferred from tree-ring cellulose δ^13^C, assuming that leaf gas-exchange information is recorded by annual growth rings (Peñuelas et al., 2011; Marchand et al., 2020). Uncertainties related to this approach include the long residence time of stemwood non-structural carbohydrates (Richardson et al., 2013), which may dampen the isotopic signal used as a proxy of gas exchanges, as well as the effect of tree size and stand age on estimated WUE (Marchand et al., 2020). Tree-ring derived WUE may also be unable to detect intra-seasonal physiological changes when tree rings are extremely narrow (i.e., mean width of about 1 mm), making it difficult to detect WUE responses to sub-monthly drought variability (Lavergne et al., 2019; but see Michelot et al., 2011; Battipaglia et al., 2014).

Tree-level WUE at timescales from days to months can be quantified through simultaneous measurements of whole-tree water use and growth using automated sap flow sensors and dendrometers (McCarthy et al., 2011). Such studies facilitate disentangling the relative role of growth and transpiration on WUE, and can, therefore, clarify physiological mechanisms underlying complicated WUE–drought relationships. For instance, Forner et al. (2018) reported an increase in WUE of *Pinus nigra* to alleviate negative effects of drought *via* restricting transpiration, but without a penalty on growth. By contrast, Sánchez-Costa et al. (2015) found that annual basal area increment was more reduced than transpiration in a dry year, resulting in a decline of tree-level WUE. In rubber plantations, WUE could possibly be regulated by factors affecting carbon sequestration rather than water consumption (Lin et al., 2018).

In the Great Basin of North America, arid conditions along the valley floors are progressively replaced by wetter and cooler environments along mountain slopes, so that high elevations environments are dominated by sky-island conifer forests that experience dry and hot summers as well as cold and snowy winters (Grayson, 2011). The Nevada Climate-ecohydrological Assessment Network (NevCAN; Mensing et al., 2013), a network of valley-to-mountain observing stations, was established in 2011 to capture climate variability and its impacts on Great Basin ecosystems. Since its inception, NevCAN data have revealed seasonal changes in atmospheric and soil variables connected with whole-tree transpiration (Liu and Biondi, 2020, 2021). We built on those previous studies to examine the linkages between tree-level WUE and seasonal drought variability for two iconic tree-line conifers, i.e., bristlecone (*Pinus longaeva* D. K. Bailey) and limber (*Pinus flexilis* E. James) pine, which showed differential responses of whole-tree transpiration (Liu and Biondi, 2020) and stem growth (Ziaco and Biondi, 2016, 2018; Ziaco et al., 2016) to seasonal drought. Our goals were (1) to determine the seasonal dynamics of tree-level WUE across years with pronounced differences in seasonal drought; and (2) to assess species-specific responses to drought impacts on tree-level WUE. To achieve the research objectives, we analyzed the sub-hourly measurements of stem size and sap flow, as well as of atmospheric and soil variables, conducted over 5 years (2013–2017).

## Materials and Methods

### Study Site and Environmental Data

Field data were collected at a subalpine NevCAN site (38°54′22″ N, 114°18′32″ W; 3,357 m a.s.l.) on the western slope of the Snake Range in central-eastern Nevada. At each NevCAN site, meteorological data are automatically recorded together with soil and tree measurements at sub-hourly intervals [see Liu and Biondi (2020, 2021), for details]. According to the public-domain version of the Parameter-Regression at Independent-Slopes Model (PRISM) dataset (Daly et al., 2008), long-term (1895–2019) mean annual temperature and total annual precipitation at the study site are 3.1 ± 0.8°C and 696 ± 160 mm, respectively. While the precipitation regime is mainly dominated by winter snowpack dynamics (77% of annual precipitation is received in October–May), summer thunderstorms are common (Mensing et al., 2013). Soils are categorized as loamy-skeletal, carbonatic Lithic Cryorthents (Johnson et al., 2014).

Sub-hourly environmental data, such as air temperature (T_*a*_, °C), precipitation (Prec, mm), relative humidity (RH,%) and soil moisture at 10 and 20 cm depth (SM_10_ and SM_20_,%) during 2013–2017 were downloaded from the NevCAN online repository^1^. Vapor pressure deficit (VPD, kPa) was calculated using 10-min records of air temperature and relative humidity as follows (Jones, 1992):


(1) $$VPD=0.611\left(1-\frac{RH}{100}\right)EXP\left(\frac{17.27\times T_{a}}{237.3+T_{a}}\right)$$

This mixed-conifer stand is dominated by bristlecone pine, limber pine, and Engelmann spruce (*Picea engelmannii* Parry ex Engelm.) with understory vegetation sparse or absent (Kilpatrick and Biondi, 2020). Five mature and healthy trees per species were randomly chosen to be instrumented with sap flow and point dendrometer sensors (Table 1). Selected limber and bristlecone pines had an average diameter at breast height (DBH) of 24.2 ± 6.9 and 65.9 ± 69.5 cm, and height of 5.2 ± 1.8 and 9.0 ± 1.2 m, respectively.

**TABLE 1:** Characteristics^*a*^ of instrumented trees at the study site.

**Tree code^*b*^**	**DBH (cm)**	**Height (m)**	***D*_*s*_ (cm)**	***A*_*s*_ (cm^2^)**	**Dendrometer sensors**	**Sap flow Sensors**
PIFL 1	17.5	4.5	3.08	139.42	1	1
PIFL 2	25.0	6.0	3.94	260.47	1	1
PIFL 3	35.0	8.0	4.75	451.15	1	1
PIFL 4	24.5	4.0	4.50	282.60	1	2
PIFL 5	19.0	3.5	2.25	118.34	1	2
PILO 6	40.0	9.0	3.35	385.48	1	1
PILO 7	30.0	10.0	2.91	247.22	1	1
PILO 8	34.0	7.0	3.30	318.11	2	2
PILO 9	35.5	9.0	3.10	315.38	2	2
PILO 10	190.0	10.0	2.65	1,558.94	1	1

*^*a*^DBH, diameter at breast height; *D*_*s*_, sapwood thickness; *A*_*s*_, sapwood area.*

*^*b*^PIFL, *Pinus flexilis* (limber pine); PILO, *Pinus longaeva* (bristlecone pine).*

Relative extractable water content (REW, unitless) in the soil, which is an indicator of soil water availability in forest stands, was used to quantify drought severity and duration. At a daily time scale, REW was defined as the ratio of actual soil water to maximum extractable soil water, as follows (Granier et al., 1999):


(2) $$REW=\left(SM-SM_{min}\right)/\left(SM_{max}-SM_{min}\right)$$

where SM_*min*_ and SM_*max*_ are the minimum and maximum soil water contents during April–October in years 2013–2017. REW varies between 0 (permanent wilting point) and 1 (field capacity). Generally, soil drought occurs with REW < 0.4, defining the threshold at which soil water availability induces stomatal closure, leading to the downregulation in transpiration for most tree species (Granier et al., 1999). Since cool-season snowpack and, therefore, deep soil water reservoir, could mitigate the drought effects on growing-season transpiration of limber pine (Liu and Biondi, 2020, 2021), we calculated the REW using the 20 cm soil moisture data. The 3-month standardized precipitation evapotranspiration index (SPEI), a multiscalar drought index that considers both precipitation and potential evapotranspiration (Vicente-Serrano et al., 2010), was used to test whether estimated REW captured seasonal and interannual variations in drought severity. Negative and positive values of SPEI indicate dry and wet conditions, respectively, and were obtained for the years 2013–2017 from the Global SPEI database^2^ using the gridded cell that includes the study site.

### Sap Flow Measurements and Whole-Tree Transpiration

Following Granier (1987), sap flux density was measured using constant thermal diffusion sensors (TDP30, Dynamax, Houston, TX, United States), mounted at breast height (∼1.3 m from the ground) and protected from rainfall, solar radiation, and physical damages by aluminum-wrapped covers. Two probes were radially inserted into the sapwood at a vertical distance of 15 cm after removing bark (Renninger et al., 2014). Each tree was equipped with one sensor on its north-facing side, and one additional sensor was placed on the south side of two trees for each species (Table 1). Temperature differences between probes were sampled every 30 s and stored as 10-min averages in an AM16/32 multiplexer (CR1000, Campbell Scientific Inc.). These signals were translated into sap flux density (*F*_*d*_, g cm^–2^ s^–1^) according to Granier’s (1987) empirically calibrated formula:


(3) $$F_{d}=0.0119\times {\left[\left(\Delta T_{max}-\Delta T\right)/\Delta T\right]}^{1.231}$$

where Δ*T* is the measured or corrected temperature difference, and Δ*T*_*max*_ is the maximum value of Δ*T* when sap flow is near zero. For three instrumented trees whose sapwood depth was < 3 cm (Table 1), Δ*T* was corrected as in our previous studies (Liu and Biondi, 2020) to eliminate the underestimation of sap flow when the probe reaches the heartwood. At high elevations, night-time vapor pressure deficit is usually low, and patterns of between-needle temperature reach equilibrium, implying that the recharge of stem water storage has completed (Wieser et al., 2014). Thus, nighttime Δ*T*_*max*_ served as a reference for the next day (Lu et al., 2004; Liu and Biondi, 2020, 2021). A species-specific calibration of the original Granier formula (Lu et al., 2004; Mei et al., 2016) was not deemed necessary because our study emphasized dynamic changes over seasons and years, and furthermore, xylem type and wood properties of our target pines resemble one of the species (*Pinus nigra*) that was used to develop the original formula.

Sapwood thickness was measured by taking two increment cores at breast height from instrumented trees in June 2013 (Table 1). Scaling sap flux density to whole-tree transpiration required estimations of cross-sectional and radial variations in sap flux density. Previous results indicated no significant differences in sap flow between two opposite sides of the same tree stem for both pine species (Liu and Biondi, 2020). Although radial patterns of sap flux density may differ (e.g., Phillips et al., 1996; Delzon et al., 2004), this was not the case for another study of high-elevation limber pine in the western United States (Fischer et al., 2002). Given that the sapwood thickness of bristlecone pine does not decrease significantly with increasing stem age in the Great Basin (Connor and Lanner, 1990), sap flows measured by 30-mm-long probes could cover 91–100% of cross-sectional sapwood area in instrumented bristlecone pines (Table 1). Therefore, whole-tree transpiration per tree per year was obtained by multiplying measured or averaged (if applicable) *F*_*d*_ by the sapwood area of both pines.

### Basal Area Increment and Tree-Level Water-Use Efficiency

Automated point dendrometer (Agricultural Electronics Corp., Tucson, AZ, United States) placed at breast height was used to continuously measure the stem radial growth of each sampled tree (Table 1). The dead outmost tissue of the bark was peeled off before sensor installation to minimize bark swelling and shrinking. Trunk radius was recorded by the linear displacements of the sensor rod, which was translated by a differential transformer into an electrical signal (Biondi and Hartsough, 2010). To allow for long-term observations, the tension of the sensing rod was adjusted when it reached the maximum measurement range of 15,000 μm. Raw data were recorded at an interval of 30 min, and records for trees equipped with two dendrometers were averaged. The daily radii for each tree were calculated and then converted to the basal area (cm^2^ d^–1^).

Coniferous species in sky-island stands of the western United States may present daily tree water deficit (i.e., stem shrinkage with zero growth; Ziaco and Biondi, 2018). Weekly stem basal area increments were, therefore, calculated to minimize the water-induced stem variations (Rossi et al., 2006). For each tree, year, and species, weekly basal area increment (BAI, cm^2^ wk^–1^) was determined following Zweifel et al. (2005), as modified by Luo et al. (2018):


(4) $$BAI=\pi \left(r_{t}^{2}-r_{t-1}^{2}\right)$$

where *r* is stem radius and *t, t_–1_* is a weekly time interval. The time interval was increased to 2–3 weeks when the calculated weekly BAI was negative, and weekly BAI was then obtained under the assumption of a constant and positive growth rate during each time interval (Luo et al., 2018). Whole-tree water-use efficiency (WUE, cm^2^ m^–3^) was obtained for each instrumented tree following Sánchez-Costa et al. (2015) as:


(5) WUE=BAI/Transpiration

where BAI is basal area increment and transpiration is the whole-tree transpiration. We computed WUE during the growing season using variables aggregated at weekly and seasonal timescales. Previous histological analysis of wood formation through repeated micro-coring of both limber and bristlecone pine had revealed that xylogenesis started in early to mid-June and ceased in early September at this site (Ziaco and Biondi, 2016; Ziaco et al., 2016). We, therefore, analyzed weekly WUE for day-of-year (DOY) 165 to 249, making the “early season” from 8 June to 31 July, and the “late season” from 1 August to 6 September.

### Data Analysis

For each calendar week during the growing season, we calculated the “baseline” WUE as its average over the 5-year study period (2013–2017). The relative change in WUE (ΔWUE,%) was then obtained as follows (Xu et al., 2019):


(6) $$\Delta WUE=100\times \left(WUE_{o}-WUE_{b}\right)/WUE_{b}$$

where WUE_*o*_ and WUE_*b*_ are weekly WUE values observed and baseline, respectively.

Repeated measures ANOVA were used to assess the effects of species, year, and their interactions on BAI, Transpiration, and WUE during the whole growing season as well as in the early- and late-season periods. Relationships of weekly BAI to weekly transpiration during the growing season as well as early- and late-season periods were estimated with simple linear models. Data pooled across species were log-10 transformed and used to test relationships between either weekly BAI or transpiration and weekly mean vapor pressure deficit or soil moisture. Differences in slopes and intercepts of linear relationships were tested by analysis of covariance (ANCOVA; McDonald, 2014). Statistical analyses were performed using SPSS 22.0 for the Windows operating system.

## Results

### Environmental and Drought Variability

**FIGURE 1:**
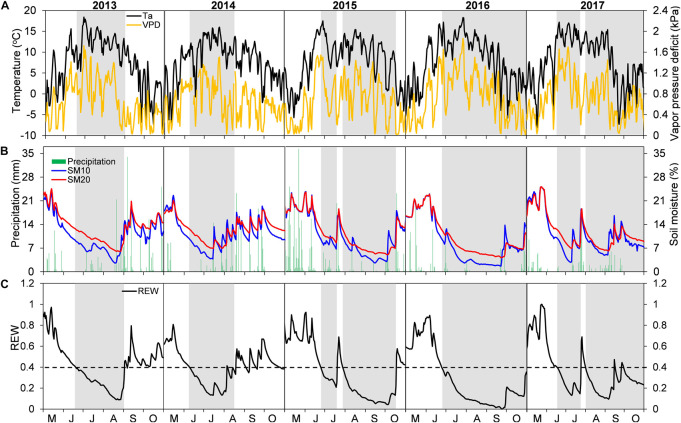
Daily variations in **(A)** mean air temperature (T_*a*_) and vapor pressure deficit (VPD), **(B)** total precipitation and soil moisture at 10 and 20 cm depths (SM_10_ and SM_20_), and **(C)** relative extractable water content (REW) at the Snake Range during May–October in years 2013–2017. Periods of drought (i.e., REW < 0.4) are portrayed as gray bands.

Interannual variations of growing-season environmental conditions were most evident when comparing early- and late-season periods (Figures 1A,B and Table 2). Early-season means T_*a*_ ranged from 10.81°C in 2015 to 12.47°C in 2016. The highest early season total precipitation and mean SM_20_ were both found in 2015, while the lowest values were recorded in 2013 and 2014, respectively. The early season means VPD varied little among years (interannual variation < 0.11 kPa). Conversely, environmental conditions during the late season in 2013–2014 were wetter and colder than those in 2015–2016, with lower mean T_*a*_ and VPD, higher total precipitation and mean SM_20_ (Table 2). Year-to-year changes of each environmental variable during the late season determined the overall interannual changes for the whole growing period (Table 2).

**TABLE 2:** Summary of environmental variables^*a*^ for the whole growing season as well as the early- and late-season at the study site during 2013–2017.

**Growing Season**	**Years**	**T_*a*_ (°C)**	**Prec (mm)**	**VPD (kPa)**	**SM_20_ (%)**
Whole	2013	10.47	158	0.78	11.18
	2014	10.06	202	0.72	11.39
	2015	10.88	112	0.84	10.38
	2016	11.05	58	0.95	8.91
	2017	10.57	109	0.82	10.05
Early	2013	12.32	25	0.99	11.57
	2014	11.26	40	0.90	9.52
	2015	10.81	95	0.85	13.97
	2016	12.47	32	1.08	12.59
	2017	12.41	70	0.98	11.14
Late	2013	8.62	133	0.58	10.79
	2014	8.86	162	0.54	13.26
	2015	10.95	17	0.83	6.80
	2016	9.63	26	0.83	5.23
	2017	8.73	39	0.67	8.96

*^*a*^T_*a*_, mean daily air temperature; Prec, total daily precipitation; VPD, mean daily vapor pressure deficit; SM_20_, mean daily soil moisture at 20-cm depth.*

Early season drought was moderate, and generally similar among years (REW > 0.2; Figure 1C), with an earlier start in 2013–2014 (early to mid-June) than in 2015–2017 (mid to late June). Late-season conditions were different between years, with drought occurring until mid to late October in 2015–2017, whereas water-stress alleviation took place in 2013–2014 during early (2014) or late (2013) August (Figure 1C). Such seasonal and interannual drought variability was also captured by the 3-month SPEI index (Supplementary Figure 1).

### Seasonal and Interannual Variations in Basal Area Increment, Transpiration, and Water-Use Efficiency

**FIGURE 2:**
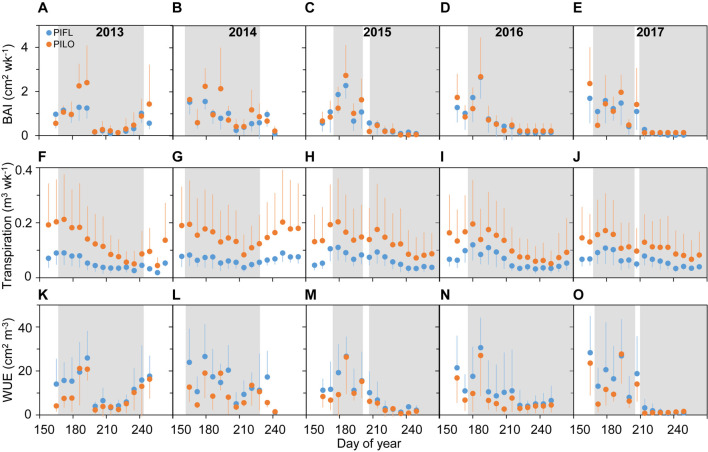
Weekly **(A–E)** basal area increment (BAI), **(F–J)** transpiration, and **(K–O)** whole-tree water-use efficiency (WUE) for limber pine (PIFL) and bristlecone pine (PILO) during the whole growing season in years 2013–2017. Gray bands represent drought periods (i.e., REW < 0.4); error bars indicate ± one standard deviation.

Limber and bristlecone pine showed different seasonal patterns of stem growth (BAI), transpiration, and water-use efficiency (Figure 2). BAI was relatively high during the early growing season, especially for bristlecone, peaking around late June to mid-July (DOY 165–187 and DOY 165–193 for limber and bristlecone pine, respectively), and then decreased to a minimum in early August (DOY 207–221; 2013–2014) or early September (DOY 249–250; 2015–2017). In 2013–2014, BAI of both species increased toward the end of the growing season thanks to abundant precipitation (cf. Figures 1B,C). Seasonal variability in transpiration was less pronounced than that of BAI, and it remained higher for bristlecone than for limber both in the early and in the late growing season (Figures 2F–J). Across species and years, WUE was relatively high (i.e., low modulation ability) throughout the early growing season (Figures 2K–O), when drought conditions were moderate. Late-growing season WUE varied between years as both species WUE increased by 211% (limber pine) and 219% (bristlecone pine) in 2013–2014, while it decreased by 89% (limber pine) and 85% (bristlecone pine) in 2015–2017 (Supplementary Figure 2).

**FIGURE 3:**
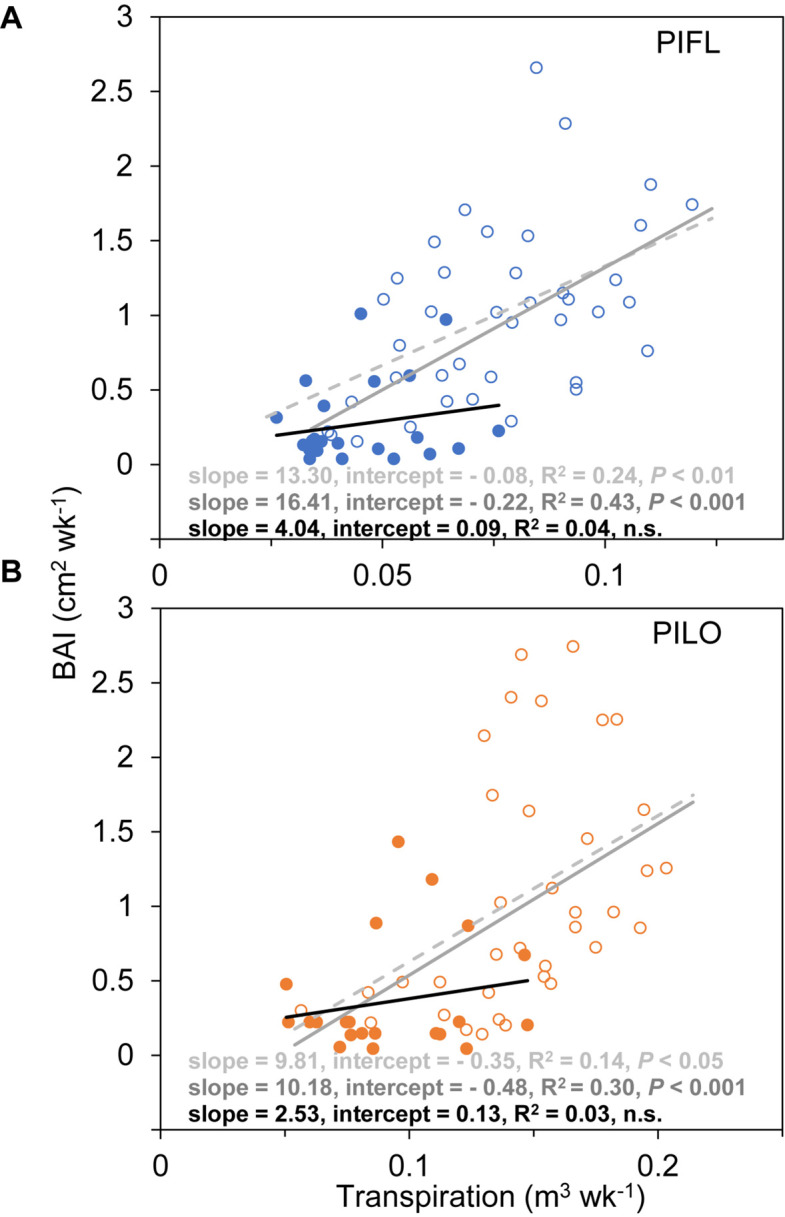
Relationships between weekly basal area increment (BAI) and whole-tree transpiration of **(A)** limber pine (PIFL), and **(B)** bristlecone pine (PILO) for the early (empty circle) and late (filled circle) growing seasons in years 2013–2017. The dashed, gray, and black solid trend lines are for the whole growing season, early-, and late-seasons, respectively.

Weekly BAI and transpiration during the early and the whole growing season were linearly correlated for both species over the 5-year period, and the BAI–transpiration relationships (slope and intercept) did not differ significantly (*P* > 0.05; Figure 3). However, the two species showed differences in WUE for the whole and the early growing season (Table 3). Limber pine was characterized by lower BAI and transpiration than bristlecone pine (Supplementary Table 1). Overall, limber had higher WUE than bristlecone pine in the whole (multi-year mean of 12.86 ± 1.51 *vs*. 8.98 ± 0.70 cm^2^ m^–3^) and in the early (multi-year mean of 16.29 ± 0.87 *vs*. 10.89 ± 0.82 cm^2^ m^–3^) growing season.

**TABLE 3:** Summary of water-use efficiency (WUE, cm^2^ m^–3^) of each tree species^*a*^ for the whole growing season as well as the early- and late-season at the study site during 2013–2017.

**Growing Season**	**Years**	**PIFL**	**PILO**	**Year**		**Species**		**Year × species**	
				***F***	***P***	***F***	***P***	***F***	***P***
Whole	2013	13.55 ± 5.01	9.35 ± 4.45	0.50	0.733	6.65	**0.014**	0.08	0.988
	2014	14.46 ± 6.25	9.47 ± 3.60						
	2015	10.43 ± 4.54	7.76 ± 4.19						
	2016	12.65 ± 6.42	9.32 ± 5.43						
	2017	13.23 ± 6.52	9.01 ± 2.47						
Early	2013	15.20 ± 7.85	9.59 ± 5.42	1.03	0.404	7.04	**0.011**	0.02	0.999
	2014	17.27 ± 7.86	10.92 ± 4.78						
	2015	15.61 ± 6.75	11.15 ± 6.17						
	2016	16.49 ± 8.68	10.96 ± 6.53						
	2017	16.88 ± 11.02	11.85 ± 4.35						
Late	2013	12.71 ± 6.20	8.88 ± 3.85	11.65	**<0.001**	1.24	0.272	0.75	0.567
	2014	10.14 ± 6.63	7.05 ± 2.61						
	2015	1.80 ± 2.74	2.12 ± 1.38						
	2016	3.95 ± 4.97	4.78 ± 2.63						
	2017	1.43 ± 1.66	1.13 ± 0.84						

*^*a*^PIFL, *Pinus flexilis* (limber pine); PILO, *Pinus longaeva* (bristlecone pine). Repeated-measures ANOVA was used to test effects of year, species, and their interaction on WUE, and differences with *P* < 0.05 are shown in bold font.*

Both species had higher late-season WUE in 2013–2014 (11.43 ± 1.82 and 7.97 ± 1.29 cm^2^ m^–3^ for limber and bristlecone pine, respectively) than in 2015–2017 (2.39 ± 1.36 and 2.68 ± 1.89 cm^2^ m^–3^ for limber and bristlecone pine, respectively) (Table 3). This difference in WUE was driven by increased stem growth without additional water use, as BAI during the late growing season in 2013–2014 was on average 3.8 (limber pine) and 3.2 (bristlecone pine) times higher than in 2015–2017, whereas transpiration changed little for both species (Supplementary Table 1). At the weekly scale, BAI was not correlated with transpiration during the late growing season (Figure 3).

### Environmental Influences on Water-Use Efficiency

**FIGURE 4:**
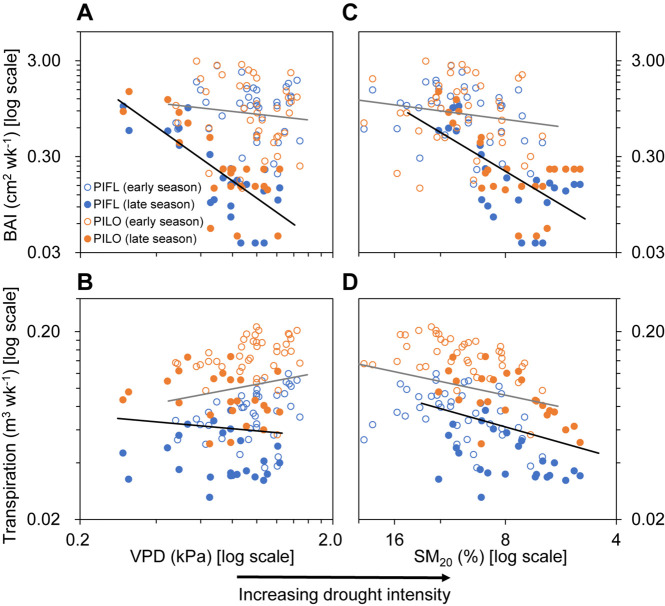
Relationships of **(A,C)** weekly basal area increment (BAI) and **(B,D)** transpiration of limber pine (PIFL) and bristlecone pine (PILO) with weekly mean vapor pressure deficit (VPD) and soil moisture at 20 cm depth (SM_20_) during the early and late growing seasons in years 2013–2017. All data were log-10 transformed. The gray and black trend lines are for the early and late growing seasons (see Supplementary Table 1), respectively.

Differences were found between the early and late growing seasons in terms of how atmospheric (VPD) or soil variables (SM_20_) influenced stem growth and whole-tree transpiration (Figure 4 and Supplementary Table 2). Correlations with BAI were low in the early season, whereas drought intensity was linked to BAI in the late one (Figures 4A,C and Supplementary Table 2). Environmental connections with late-season transpiration were similar to those with BAI, albeit less pronounced (Figures 4B,D and Supplementary Table 2) and not significantly different between the early and late growing seasons (Supplementary Table 2).

**FIGURE 5:**
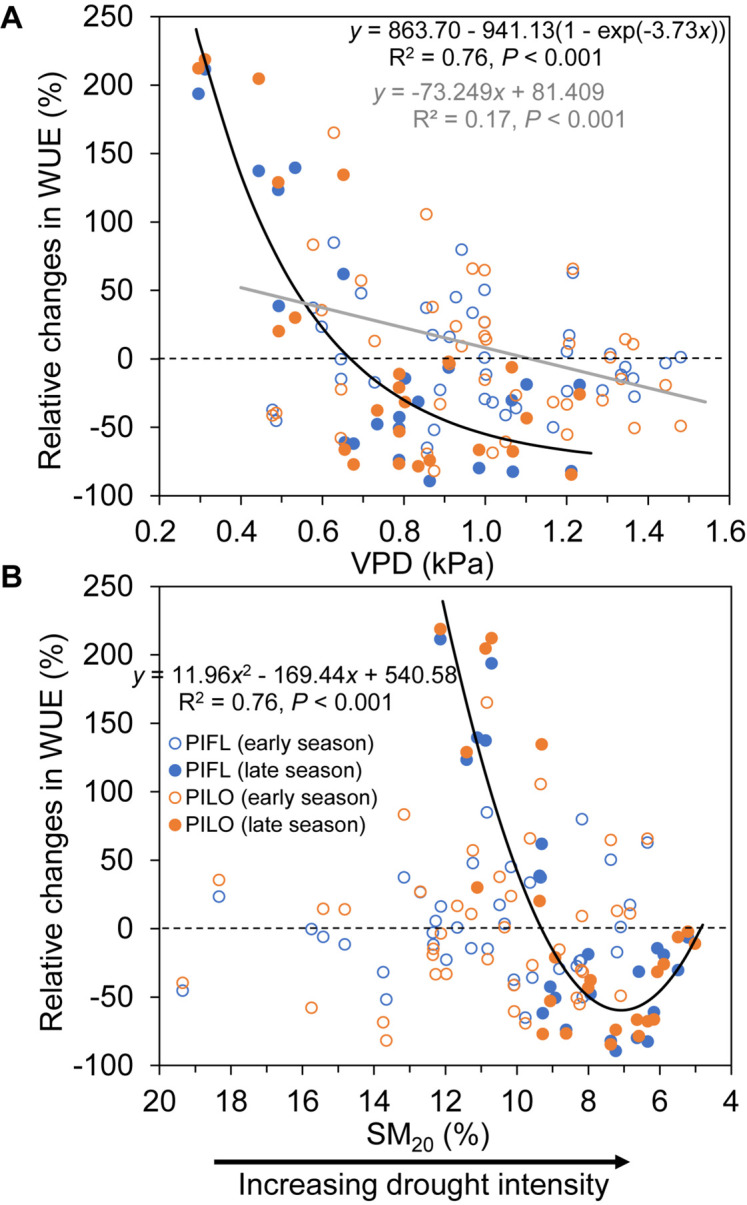
Relationships between relative changes in water-use efficiency (ΔWUE; see text for details) of limber pine (PIFL) and bristlecone pine (PILO) and **(A)** vapor pressure deficit (VPD), and **(B)** soil moisture at 20-cm depth (SM_20_) for the early (gray trend line) and late (black trend line) growing seasons in years 2013–2017.

Relative changes in WUE also exhibited a different pattern between the early and late growing seasons (Figure 5). During the early season, a linear relationship between relative changes in WUE and VPD explained 17% of variance, so that relative changes in WUE were positive when VPD was < 1 kPa, and became negative for higher VPD values. In contrast, relative changes in WUE were non-linearly related to either VPD or SM_20_ during the late season. As shown in Figure 5, ΔWUE dropped quickly either for VPD < 1 kPa or for SM_20_ > 7%, but it slightly increased again under the extreme soil drought (SM_20_ < 7%).

## Discussion

### Differences in Water-Use Efficiency Between Species

Whole-tree WUE, when derived from concurrent measurements of stem radius and sap flow, can provide a useful metric for quantifying species differences for optimizing tree growth in water-limited environments (McCarthy et al., 2011). Growing-season WUE for two Great Basin sky-island pines (Table 3) fell within the range of 2.31–14.60 cm^2^ m^–3^ reported for other pine species in Mediterranean forests (Sánchez-Costa et al., 2015; Forner et al., 2018). Furthermore, the growing-season WUE we calculated were generally higher than WUE of broadleaf species in Mediterranean forests (0.26–11.3 cm^2^ m^–3^; Sánchez-Costa et al., 2015; Nadal-Sala et al., 2017; Forner et al., 2018) and in California urban ecosystems (0.11–7.0 cm^2^ m^–3^; McCarthy et al., 2011).

Limber pine showed higher WUE compared to bristlecone pine independent of the year during both the whole and the early growing season (Figure 2 and Table 3). There are small differences in sapwood density between Great Basin bristlecone and limber pines (0.48 and 0.41 g cm^–3^, respectively; Bentz et al., 2017), and lumen area of wood formed in the same years is smaller in bristlecone than in co-occurring limber pine (Ziaco et al., 2014). Bristlecone pine tends to invest relatively more resources into defense traits (resin ducts and constitutive monoterpenes) than limber pine, making it less vulnerable to outbreaks of mountain pine beetle and subsequent tree mortality (Bentz et al., 2017). The observed between-species difference in WUE suggests that bristlecone and limber pines, when found together in sky-island ecosystems of the Great Basin, may adopt different degrees in structural and/or physiological coordination at a whole-plant level to withstand drought stress. For instance, Shemesh et al. (2020) found that limber seedlings are favored over bristlecone seedlings because of symbiotic mycorrhizal formations, and such root-level processes may also be responsible for higher WUE in mature limber pines compared to co-occurring bristlecones.

### Effects of Seasonal Drought Variability on Water-Use Efficiency

Our *in situ* observations captured years of opposite-sign precipitation anomalies during cold- and warm-seasons (Figure 1 and Table 2; see also Liu and Biondi, 2020). Although the dry winter and spring in 2013–2014 led to an early beginning of summer drought compared to the wetter winter and spring in 2015–2017, the early season drought intensity was almost identical and moderate among years (REW > 0.2; Figure 1C). During the late growing season, however, the interannual variability of summer rains resulted in drought relief for 2013–2014 (i.e., REW > 0.4) and enhanced drought severity for 2015–2017 (REW < 0.2; Figure 1C). Semi-arid forests actively adjust their carbon and water metabolisms at either ecosystem (e.g., Knowles et al., 2020) or tree levels (e.g., Ziaco et al., 2018; Liu and Biondi, 2020) following changes in precipitation seasonality. We found that both pines were able to maintain high WUE with limited modulation ability during the early growing season regardless of the interannual variations in the timing of early season drought duration and intensity, whereas they showed high interannual WUE plasticity to deal with enhanced dry conditions or wet extremes during the late season (Figures 2K–O, Supplementary Figure 2, and Table 3). WUE of another pine species (*Pinus nigra*) adapted to water-limited environments also responded to changing seasonal drought conditions (Forner et al., 2018).

The high WUE in the early growing season was primarily driven by BAI rather than transpiration, which showed limited seasonal variability compared to BAI (Figures 2A–J). High-level WUE of California urban tree species also occurred and was maintained throughout the early season (McCarthy et al., 2011). During the early growing season, we found synchronous drought response of tree growth and transpiration (Figure 4 and Supplementary Table 2), which were thereby closely linked for both pines (Figure 3). The slopes of the BAI–transpiration relationship mirrored multi-year averaged WUE in the early and whole growing seasons, and the coupling between tree growth and transpiration could underlie the fact that the two pines did not modulate early season WUE. Possibly for the same reason, tree-ring-derived intrinsic WUE of one high elevation spruce species in semi-arid areas of western China (Wu et al., 2015) and of three conifer species in the Rocky Mountains (Marshall and Monserud, 1996) remained stable over multiple years despite climate warming and increasing atmospheric CO_2_ concentrations. It is possible that parallel adjustments of stem growth and water use occur in sky-island conifers under the moderate early season drought.

A non-significant relationship between BAI and transpiration was found in the late growing season (Figure 3). The occurrence of BAI–transpiration decoupling for late-season WUE was also reported by Forner et al. (2018) for *Pinus nigra* in a Mediterranean forest. That species WUE increased under drought by modulating water consumption more than stem growth (Forner et al., 2018), whereas bristlecone and limber pines displayed higher sensitivities for tree growth compared to transpiration during the late growing season (Figure 4 and Supplementary Table 2). The processes involved in wood formation are often more responsive than photosynthesis/transpiration to drought or rewetting (Hsiao and Acevedo, 1974; Muller et al., 2011; Körner, 2015) because xylem cell division and enlargement are physiologically driven by cell turgor (Steppe et al., 2015). Cellular or tree-level measurements have demonstrated that high late-season water stress would induce near-zero growth or trigger wood formation cessation through a critical plant water potential (Lempereur et al., 2015; Ziaco and Biondi, 2016; Cabon et al., 2020; Zhang et al., 2021). Improvement in late-season moisture conditions could then reactivate cambial activity, usually promoting the formation of false rings (Camarero et al., 2010; Ziaco et al., 2018), which were, however, absent in the two species we analyzed.

Complete stomatal closure does not seem to occur in limber and bristlecone pines even under late-season drought, as they usually show limited post-drought transpiration recovery after rewetting events (Liu and Biondi, 2020, 2021). Growth-dominated WUE variability thus drove late-season WUE of both pines in response to dry conditions in 2015–2017 and to wet ones in 2013–2014 (Figure 2 and Supplementary Figure 2). One could then hypothesize that late-season environmental conditions override species effects on WUE (Table 3). Also, the higher sensitivity of stem growth than transpiration to late-season drought suggests that transpiration would decrease even when near-zero growth was reached, placing the drought threshold for stomatal closure below that for nil stem growth (Lempereur et al., 2015). This, in turn, may be responsible for the slight increase in WUE under the extreme soil drought (SM_20_ < 7%).

Although plants usually mitigate water stress by increasing WUE through stomatal closure (Beer et al., 2009), our results suggested that late-season drought could diminish the resistance of sky-island pine species to drought (i.e., WUE declined). This tree-level behavior replicated the ecosystem-scale observations from different hydroclimatic conditions and biome types in northern China (Xu et al., 2019) and from a boreal Scots pine forest in Finland (Gao et al., 2017), where WUE decreased under water deficit or soil droughts by influencing the stomatal optimum (Yang et al., 2010). Nevertheless, both pines also increased WUE when drought relief occurred during the late growing season. Such improvement in WUE indicates that semi-arid coniferous species can withstand drought-related physiological stress and retain their early season sensitivity of stem growth to soil water availability (Ponce-Campos et al., 2013). Our multi-year analyses, therefore, revealed vapor pressure deficit and soil moisture thresholds that led to opposite responses of WUE to late-season dry or wet conditions (Figure 5), which should help with evaluating tree resistance and resilience to climate anomalies in water-limited environments.

## Conclusion

Seasonal dynamics of tree-level WUE for two co-existing sky-island pine species were linked with atmospheric and soil indicators of drought variability. Bristlecone pine presented a lower WUE than limber pine, suggesting that coexisting Great Basin pines may employ different strategies to withstand drought stress. Both pines maintained relatively high WUE during the early growing season every year due to coupling between transpiration and BAI. However, both pines modulated late-season WUE showed higher drought response of tree growth than transpiration. This behavior suggests that stem growth plays a central role in controlling late-season WUE. Dry and wet conditions influenced the late-season WUE differently when soil moisture and vapor pressure deficit reached specific values. This study advances our understanding of tree-level WUE and its association with seasonal drought variability, which helps with designing appropriate management strategies and reduces the uncertainties associated with the impact of future climatic changes. Given the iconic status of the sky-island ecosystems we studied, our results have a direct connection with designing science-driven best-management conservation strategies specifically tailored to such fascinating areas in a changing world.

## Data Availability Statement

The raw data supporting the conclusions of this article will be made available by the authors, without undue reservation.

## Author Contributions

XL originally formulated the idea and developed methodology, performed the data analyses, and wrote a draft of the manuscript. EZ and FB conducted the fieldwork. EZ provided editorial advice. FB fully revised and finalized the manuscript. All authors contributed to the article and approved the submitted version.

## Author Disclaimer

The views and conclusions contained in this manuscript are those of the authors and should not be interpreted as representing the opinions or policies of the funding agencies and supporting institutions.

## Conflict of Interest

The authors declare that the research was conducted in the absence of any commercial or financial relationships that could be construed as a potential conflict of interest.

## Publisher’s Note

All claims expressed in this article are solely those of the authors and do not necessarily represent those of their affiliated organizations, or those of the publisher, the editors and the reviewers. Any product that may be evaluated in this article, or claim that may be made by its manufacturer, is not guaranteed or endorsed by the publisher.

## Footnotes

1
ftp://sensor.nevada.edu/

2
https://spei.csic.es/database.html

## References

[B1] Battipaglia G. De Micco V. Brand W. A. Saurer M. Aronne G. Linke P. (2014). Drought impact on water use efficiency and intra-annual density fluctuations in *Erica arborea* on Elba (Italy). *Plant Cell Environ.* 37 382–391. 10.1111/pce.12160 23848555

[B2] Beer C. Ciais P. Reichstein M. Baldocchi D. Law B. E. Papale D. (2009). Temporal and among-site variability of inherent water use efficiency at the ecosystem level. *Glob. Biogeochem. Cycles* 23:GB2018. 10.1029/2008GB003233

[B3] Bentz B. J. Hood S. M. Hansen E. M. Vandygriff J. C. Mock K. E. (2017). Defense traits in the long-lived Great Basin bristlecone pine and resistance to the native herbivore mountain pine beetle. *New Phytol.* 213 611–624. 10.1111/nph.14191 27612209 PMC5213150

[B4] Biondi F. Hartsough P. C. (2010). Using automated point dendrometers to analyze tropical treeline stem growth at Nevado de Colima, Mexico. *Sensors* 10 5827–5844. 10.3390/s100605827 22219689 PMC3247734

[B5] Cabon A. Peters R. L. Fonti P. Martínez-Vilalta J. Cáceres M. D. (2020). Temperature and water potential co-limit stem cambial activity along a steep elevational gradient. *New Phytol.* 226 1325–1340. 10.1111/nph.16456 31998968

[B6] Camarero J. J. Olano J. M. Parras A. (2010). Plastic bimodal xylogenesis in conifers from continental Mediterranean climates. *New Phytol.* 185 471–480. 10.1111/j.1469-8137.2009.03073.x 19895415

[B7] Connor K. F. Lanner R. M. (1990). Effects of tree age on secondary xylem and phloem anatomy in stems of Great Basin bristlecone pine (*Pinus longaeva*). *Am. J. Bot.* 77 1070–1077. 10.1002/j.1537-2197.1990.tb13602.x

[B8] Daly C. Halbleib M. Smith J. I. Gibson W. P. Doggett M. K. Taylor G. H. (2008). Physiographically sensitive mapping of temperature and precipitation across the conterminous United States. *Int. J. Climatol.* 28 2031–2064. 10.1002/joc.1688

[B9] del Campo A. D. Sonzález-Sanchis M. Lidón A. García-Prats A. Lull C. Bautista I. (2017). “Ecohydrological-based forest management in semi-arid climate,” in *Ecosystem Services of Headwater Catchments*, eds Křeček J. Haigh M. Hofer T. Kubin E. Promper C. (Cham: Springer).

[B10] Delzon S. Sartore M. Granier A. Loustau D. (2004). Radial profiles of sap flow with increasing tree size in maritime pine. *Tree. Physiol.* 24 1285–1293. 10.1093/treephys/24.11.1285 15339738

[B11] Driscoll A. W. Bitter N. Q. Sandquist D. R. Ehleringer J. R. (2020). Multidecadal records of intrinsic water-use efficiency in the desert shrub *Encelia farinosa* reveal strong responses to climate change. *Proc. Natl. Acad. Sci. U S A.* 117 18161–18168. 10.1073/pnas.2008345117 32719142 PMC7414048

[B12] Fischer D. G. Kolb T. E. DeWald L. E. (2002). Changes in whole-tree water relations during ontogeny of *Pinus flexilis* and *Pinus ponderosa* in a high-elevation meadow. *Tree Physiol.* 22 675–685. 10.1093/treephys/22.10.675 12091149

[B13] Forner A. Valladares F. Bonal D. Granier G. Grossiord C. Aranda I. (2018). Extreme droughts affecting Mediterranean tree species’ growth and water-use efficiency: the importance of timing. *Tree Physiol.* 38 1127–1137. 10.1093/treephys/tpy022 29554342

[B14] Frank D. Reichstein M. Bahn M. Thonicke K. Frank D. Mahecha M. D. (2015). Effects of climate extremes on the terrestrial carbon cycle: concepts, processes and potential future impacts. *Glob. Chang. Biol.* 21 2861–2880. 10.1111/gcb.12916 25752680 PMC4676934

[B15] Gao Y. Markkanen T. Aurela M. Mammarella I. Thum T. Tsuruta A. (2017). Response of water use efficiency to summer drought in a boreal Scots pine forest in Finland. *Biogeosciences* 14 4409–4422. 10.5194/bg-14-4409-2017

[B16] Granier A. (1987). Evaluation of transpiration in a Douglas-fir stand by means of sap flow measurements. *Tree Physiol.* 3 309–320. 10.1093/treephys/3.4.309 14975915

[B17] Granier A. Bréda N. Biron P. Villette S. (1999). A lumped water balance model to evaluate duration and intensity of drought constraints in forest stands. *Ecol. Model.* 116 269–283. 10.1016/s0304-3800(98)00205-1

[B18] Grayson D. K. (2011). *The Great Basin: A Natural Prehistory.* Berkeley: University of California Press.

[B19] Hartmann H. Trumbore S. (2016). Understanding the roles of nonstructural carbohydrates in forest trees – from what we can measure to what we want to know. *New Phytol.* 211 386–403. 10.1111/nph.13955 27061438

[B20] Hsiao T. C. Acevedo E. (1974). Plant responses to water deficits, water-use efficiency, and drought resistance. *Agric. Meteorol.* 14 59–84. 10.1016/B978-0-444-41273-7.50012-X

[B21] Johnson B. G. Verburg P. S. J. Arnone J. A. III (2014). Effects of climate and vegetation on soil nutrients and chemistry in the Great Basin studied along a latitudinal-elevational climate gradient. *Plant Soil* 382 151–163. 10.1007/s11104-014-2144-3

[B22] Jones H. G. (1992). *Plants and microclimate: a quantitative approach to environmental plant physiology*, 2nd Edn. Cambridge: Cambridge University Press.

[B23] Keenan T. F. Hollinger D. Y. Bohrer G. Dragoni D. Munger J. W. Schmid H. P. (2013). Increase in forest water-use efficiency as atmospheric carbon dioxide concentrations rise. *Nature* 499 324–327. 10.1038/nature12291 23842499

[B24] Kilpatrick M. Biondi F. (2020). Post-Wildfire Regeneration in a Sky-Island Mixed-Conifer Ecosystem of the North American Great Basin. *Forests* 11:900. 10.3390/f11090900

[B25] Knowles J. F. Scott R. L. Minor R. L. Barron-Gafford G. A. (2020). Ecosystem carbon and water cycling from a sky island montane forest. *Agric. For. Meteorol.* 281:107835. 10.1016/j.agrformet.2019.107835

[B26] Körner C. (2015). Paradigm shift in plant growth control. *Curr. Opin. Plant Biol.* 25 107–114. 10.1016/j.pbi.2015.05.003 26037389

[B27] Lavergne A. Graven H. De Kauwe M. G. Keenan T. F. Medlyn B. E. Prentice I. C. (2019). Observed and modelled historical trends in the water-use efficiency of plants and ecosystems. *Glob. Chang. Biol.* 25 2242–2257. 10.1111/gcb.14634 30933410

[B28] Law B. E. Falge E. Gu L. Baldocchi D. D. Bakwin P. Berbigier P. (2002). Environmental controls over carbon dioxide and water vapor exchange of terrestrial vegetation. *Agric. For. Meteorol.* 113 97–120. 10.1016/S0168-1923(02)00104-1

[B29] Lempereur M. Martin-StPaul N. K. Damesin C. Joffre R. Ourcival J. Rocheteau A. (2015). Growth duration is a better predictor of stem increment than carbon supply in a Mediterranean oak forest: implications for assessing forest productivity under climate change. *New Phytol.* 207 579–590. 10.1111/nph.13400 25913661

[B30] Lin Y. Grace J. Zhao W. Dong Y. Zhang X. Zhou L. (2018). Water-use efficiency and its relationship with environmental and biological factors in a rubber plantation. *J. Hydrol.* 563 273–282. 10.1016/j.jhydrol.2018.05.026

[B31] Linares J. C. Camarero J. J. (2012). From pattern to process: linking intrinsic water-use efficiency to drought-induced forest decline. *Glob. Chang. Biol.* 18 1000–1015. 10.1111/j.1365-2486.2011.02566.x

[B32] Liu X. Biondi F. (2020). Transpiration drivers of high-elevation five-needle pines (*Pinus longaeva* and *Pinus flexilis*) in sky-island ecosystems of the North American Great Basin. *Sci. Total Environ.* 739:139861. 10.1016/j.scitotenv.2020.139861 32544678

[B33] Liu X. Biondi F. (2021). Inter-specific transpiration differences between aspen, spruce, and pine in a sky-island ecosystem of the North American Great Basin. *For. Ecol. Manage.* 491:119157. 10.1016/j.foreco.2021.119157

[B34] Loehle S. Idso C. Wigley T. B. (2016). Physiological and ecological factors influencing recent trends in United States forest health responses to climate change. *For. Ecol. Manage.* 363 179–189. 10.1016/j.foreco.2015.12.042

[B35] López R. Cano F. J. Rodríguez-Calcerrada J. Sangüesa-Barreda G. Gazol A. Camarero J. J. (2021). Tree-ring density and carbon isotope composition are early-warning signals of drought-induced mortality in the drought tolerant Canary Island pine. *Agric. For. Meteorol.* 310:108634. 10.1016/j.agrformet.2021.108634

[B36] Lu P. Urban L. Zhao P. (2004). Granier’s Thermal Dissipation Probe (TDP) Method for Measuring Sap Flow in Trees: Theory and Practice. *Acta Bot. Sin.* 46 631–646.

[B37] Luo T. Liu X. Zhang L. Li X. Pan Y. Wright I. J. (2018). Summer solstice marks a seasonal shift in temperature sensitivity of stem growth and nitrogen-use efficiency in cold-limited forests. *Agric. For. Meteorol.* 248 469–478. 10.1016/j.agrformet.2017.10.029

[B38] Marchand W. Girardin M. P. Hartmann H. Depardieu C. Isabel N. Gauthier S. (2020). Strong overestimation of water-use efficiency responses to rising CO_2_ in tree-ring studies. *Glob. Chang. Biol.* 26 4538–4558. 10.1111/gcb.15166 32421921

[B39] Marshall J. D. Monserud R. A. (1996). Homeostatic gas-exchange parameters inferred from 13 C/12 C in tree rings of conifers. *Oecologia* 105 13–21. 10.1007/BF00328786 28307117

[B40] McCarthy H. R. Pataki D. E. Jenerette G. D. (2011). Plant water-use efficiency as a metric of urban ecosystem services. *Ecol. Appl.* 21 3115–3127. 10.1890/11-0048.1

[B41] McDonald J. H. (2014). *Handbook of Biological Statistics*, edn. 3 Edn. Baltimore, MD: Sparky House Publishing.

[B42] Mei T. Fang D. Röll A. Niu F. Hendrayanto H. D. (2016). Water Use Patterns of Four Tropical Bamboo Species Assessed with Sap Flux Measurements. *Front. Plant Sci.* 6:1202. 10.3389/fpls.2015.01202 26779233 PMC4703849

[B43] Mensing S. Strachan S. Arnone J. Fenstermaker L. Biondi F. Devitt D. (2013). A network for observing Great Basin climate change. *Eos Trans. Am. Geophys. Union* 94 105–106. 10.1002/2013EO110001

[B44] Michelot A. Eglin T. Dufrêne E. Lelarge-Trouverie C. Damesin C. (2011). Comparison of seasonal variations in water-use efficiency calculated from the carbon isotope composition of tree rings and flux data in a temperate forest. *Plant Cell Environ.* 34 230–244. 10.1111/j.1365-3040.2010.02238.x 20955221

[B45] Monson R. K. Prater M. R. Hu J. Burns S. P. Sparks J. P. Sparks K. L. (2010). Tree species effects on ecosystem water-use efficiency in a high-elevation, subalpine forest. *Oecologia* 162 491–504. 10.1007/s00442-009-1465-z 19784850

[B46] Muller B. Pantin F. Génard M. Turc O. Freixes S. Piques M. (2011). Water deficits uncouple growth from photosynthesis, increase C content, and modify the relationships between C and growth in sink organs. *J. Exp. Bot.* 62 1715–1729. 10.1093/jxb/erq438 21239376

[B47] Nadal-Sala D. Sabaté S. Sánchez-Costa E. Poblador S. Sabater F. Gracia C. (2017). Growth and water use performance of four co-occurring riparian tree species in a Mediterranean riparian forest. *For. Ecol. Manage.* 396 132–142. 10.1016/j.foreco.2017.04.021

[B48] Peñuelas J. Canadell J. G. Ogaya R. (2011). Increased water-use efficiency during the 20th century did not translate into enhanced tree growth. *Glob. Ecol. Biogeogr.* 20 597–608. 10.1111/j.1466-8238.2010.00608.x

[B49] Phillips N. Oren R. Zimmermann R. (1996). Radial patterns of xylem sap flow in non-, diffuse- and ring-porous tree species. *Plant Cell Environ.* 19 983–990. 10.1111/j.1365-3040.1996.tb00463.x

[B50] Ponce-Campos G. E. Moran M. S. Huete A. R. Zhang Y. Bresloff C. Huxman T. E. (2013). Ecosystem resilience despite large-scale altered hydroclimatic conditions. *Nature* 494 349–352. 10.1038/nature11836 23334410

[B51] Renninger H. J. Carlo N. Clark K. L. Schäfer K. V. R. (2014). Physiological strategies of co-occurring oaks in a water- and nutrient-limited ecosystem. *Tree Physiol.* 34 159–173. 10.1093/treephys/tpt122 24488856

[B52] Richardson A. D. Carbone M. S. Keenan T. F. Czimczik C. I. Hollinger D. Y. Murakami P. (2013). Seasonal dynamics and age of stemwood nonstructural carbohydrates in temperate forest trees. *New Phytol.* 197 850–861. 10.1111/nph.12042 23190200

[B53] Rossi S. Deslauriers A. Anfodillo T. Morin H. Saracino A. Motta R. (2006). Conifers in cold environments synchronize maximum growth rate of tree-ring formation with day length. *New Phytol.* 170 301–310. 10.1111/j.1469-8137.2006.01660.x 16608455

[B54] Sánchez-Costa E. Poyatos R. Sabaté S. (2015). Contrasting growth and water use strategies in four co-occurring Mediterranean tree species revealed by concurrent measurements of sap flow and stem diameter variations. *Agric. For. Meteorol.* 207 24–37. 10.1016/j.agrformet.2015.03.012

[B55] Shemesh H. Boaz B. E. Millar C. I. Bruns T. D. (2020). Symbiotic interactions above treeline of long-lived pines: Mycorrhizal advantage of limber pine (*Pinus flexilis*) over Great Basin bristlecone pine (*Pinus longaeva*) at the seedling stage. *J. Ecol.* 108 908–916. 10.1111/1365-2745.13312

[B56] Steppe K. Sterck F. Deslauriers A. (2015). Diel growth dynamics in tree stems: linking anatomy and ecophysiology. *Trends Plant Sci.* 20 335–343. 10.1016/j.tplants.2015.03.015 25911419

[B57] Vicente-Serrano S. M. Beguería S. López-Moreno J. I. (2010). A multiscalar drought index sensitive to global warming: the standardized precipitation evapotranspiration index. *J. Climate* 23 1696–1718. 10.2307/26189715

[B58] Wieser G. Gruber A. Oberhuber W. (2014). Sap flow characteristics and whole-tree water use of *Pinus cembra* across the treeline ecotone of the central Tyrolean Alps. *Eur. J. For. Res.* 133 287–295. 10.1007/s10342-013-0760-8

[B59] Wu G. Liu X. Chen T. Xu G. Wang W. Zeng X. (2015). Elevation-dependent variations of tree growth and intrinsic water-use efficiency in Schrenk spruce (*Picea schrenkiana*) in the western Tianshan Mountains, China. *Front. Plant Sci.* 6:309. 10.3389/fpls.2015.00309 25999973 PMC4422019

[B60] Xu H. Wang X. Zhao C. Zhang X. (2019). Responses of ecosystem water use efficiency to meteorological drought under different biomes and drought magnitudes in northern China. *Agric. For. Meteorol.* 278:107660. 10.1016/j.agrformet.2019.107660

[B61] Yang B. Pallardy S. G. Meyers T. P. Gu L. Hanson P. J. Wullschleger S. D. (2010). Environmental controls on water use efficiency during severe drought in an Ozark Forest in Missouri, USA. *Glob. Chang. Biol.* 16 2252–2271. 10.1111/j.1365-2486.2009.02138.x

[B62] Yi K. Maxwell J. T. Wenzel M. K. Roman D. T. Sauer P. E. Phillips R. P. (2018). Linking variation in intrinsic water-use efficiency to isohydricity: a comparison at multiple spatiotemporal scales. *New Phytol.* 221 195–208. 10.1111/nph.15384 30117538

[B63] Zhang J. Gou X. Alexander M. R. Xia J. Wang F. Zhang F. (2021). Drought limits wood production of *Juniperus przewalskii* even as growing seasons lengthens in a cold and arid environment. *Catena* 196:104936. 10.1016/j.catena.2020.104936

[B64] Ziaco E. Biondi F. (2016). Tree growth, cambial phenology, and wood anatomy of limber pine at a Great Basin (USA) mountain observatory. *Trees* 30 1507–1521. 10.1007/s00468-016-1384-7

[B65] Ziaco E. Biondi F. (2018). Stem Circadian Phenology of Four Pine Species in Naturally Contrasting Climates from Sky-Island Forests of the Western USA. *Forests* 9:396. 10.3390/f9070396

[B66] Ziaco E. Biondi F. Rossi S. Deslauriers A. (2014). Intra-annual wood anatomical features of high-elevation conifers in the Great Basin, USA. *Dendrochronologia* 32 303–312. 10.1016/j.dendro.2014.07.006

[B67] Ziaco E. Biondi F. Rossi S. Deslauriers A. (2016). Environmental drivers of cambial phenology in Great Basin bristlecone pine. *Tree Physiol.* 36 818–831. 10.1093/treephys/tpw006 26917705

[B68] Ziaco E. Truettner C. Biondi F. Bullock S. (2018). Moisture-driven xylogenesis in *Pinus ponderosa* from a Mojave Desert mountain reveals high phenological plasticity. *Plant Cell Environ.* 41 823–836. 10.1111/pce.13152 29361193

[B69] Zweifel R. Zimmermann L. Newbery D. M. (2005). Modeling tree water deficit from microclimate: an approach to quantifying drought stress. *Tree Physiol.* 25 147–156. 10.1093/treephys/25.2.147 15574396

