# Supplementary material for: Water-Use Efficiency of Co-occurring Sky-Island Pine Species in the North American Great Basin
Source: Front Plant Sci. 2021 Dec 3;12:787297. doi: 10.3389/fpls.2021.787297 (PMC8678526; doi:10.3389/fpls.2021.787297)
Supplement: Supplementary file 1 [file Data_Sheet_1.zip › Liu et al. 2021 Supplement..docx]

**Supplementary information: Tables S1-S2 and Figures S1-S2.**

**Supplementary Table S1:** Summary of basal area increment (BAI, cm^2^) and transpiration (*T,* m^3^) of each tree species^a^ for the whole growing season as well as the early- and late-season at the study site during 2013–2017. Repeated-measures ANOVA was used to tests effects of year, species, and their interaction on those variables, and differences with *P* < 0.05 are shown in bold font.

|  | Growing Season | Years | PIFL | PILO |  | Year | |  | Species | |  | Year × species | |
| --- | --- | --- | --- | --- | --- | --- | --- | --- | --- | --- | --- | --- | --- |
|  |  |  |  |  |  | *F* | *P* |  | *F* | *P* |  | *F* | *P* |
| BAI | Whole | 2013 | 8.29 ± 1.25 | 11.44 ± 2.16 |  | 0.92 | 0.464 |  | 4.79 | **0.035** |  | 0.46 | 0.766 |
|  |  | 2014 | 8.93 ± 1.74 | 13.03 ± 2.74 |  |  |  |  |  |  |  |  |  |
|  |  | 2015 | 8.72 ± 1.82 | 9.26 ± 2.75 |  |  |  |  |  |  |  |  |  |
|  |  | 2016 | 9.41 ± 1.80 | 10.01 ± 1.91 |  |  |  |  |  |  |  |  |  |
|  |  | 2017 | 9.94 ± 1.61 | 10.49 ± 3.88 |  |  |  |  |  |  |  |  |  |
|  | Early | 2013 | 5.98 ± 1.30 | 7.67 ± 1.37 |  | 3.54 | 0.067 |  | 3.26 | **0.021** |  | 0.75 | 0.566 |
|  |  | 2014 | 6.27 ± 0.30 | 9.27 ± 3.24 |  |  |  |  |  |  |  |  |  |
|  |  | 2015 | 8.06 ± 1.35 | 8.37 ± 2.05 |  |  |  |  |  |  |  |  |  |
|  |  | 2016 | 8.57 ± 1.41 | 8.17 ± 1.00 |  |  |  |  |  |  |  |  |  |
|  |  | 2017 | 8.47 ± 1.79 | 9.72 ± 6.96 |  |  |  |  |  |  |  |  |  |
|  | Late | 2013 | 2.31 ± 0.66 | 3.78 ± 3.52 |  | 5.81 | **0.001** |  | 3.30 | 0.08 |  | 0.28 | 0.89 |
|  |  | 2014 | 2.66 ± 2.12 | 3.77 ± 2.19 |  |  |  |  |  |  |  |  |  |
|  |  | 2015 | 0.66 ± 0.89 | 0.90 ± 0.74 |  |  |  |  |  |  |  |  |  |
|  |  | 2016 | 0.84 ± 1.06 | 1.83 ± 1.75 |  |  |  |  |  |  |  |  |  |
|  |  | 2017 | 0.46 ± 0.51 | 0.77 ± 0.80 |  |  |  |  |  |  |  |  |  |
| *T* | Whole | 2013 | 0.67 ± 0.24 | 1.61 ± 0.80 |  | 0.08 | 0.988 |  | 8.40 | **0.006** |  | 0.06 | 0.994 |
|  |  | 2014 | 0.74 ± 0.29 | 1.73 ± 0.97 |  |  |  |  |  |  |  |  |  |
|  |  | 2015 | 0.93 ± 0.35 | 1.84 ± 0.97 |  |  |  |  |  |  |  |  |  |
|  |  | 2016 | 0.86 ± 0.34 | 1.52 ± 0.86 |  |  |  |  |  |  |  |  |  |
|  |  | 2017 | 0.88 ± 0.37 | 1.56 ± 0.84 |  |  |  |  |  |  |  |  |  |
|  | Early | 2013 | 0.47 ± 0.22 | 1.16 ± 0.95 |  | 0.07 | 0.992 |  | 8.59 | **0.006** |  | 0.09 | 0.986 |
|  |  | 2014 | 0.47 ± 0.22 | 1.10 ± 0.92 |  |  |  |  |  |  |  |  |  |
|  |  | 2015 | 0.58 ± 0.23 | 1.12 ± 0.91 |  |  |  |  |  |  |  |  |  |
|  |  | 2016 | 0.64 ± 0.31 | 1.10 ± 0.90 |  |  |  |  |  |  |  |  |  |
|  |  | 2017 | 0.55 ± 0.25 | 0.93 ± 0.75 |  |  |  |  |  |  |  |  |  |
|  | Late | 2013 | 0.20 ± 0.06 | 0.45 ± 0.39 |  | 0.62 | 0.649 |  | 7.45 | **0.009** |  | 0.09 | 0.985 |
|  |  | 2014 | 0.27 ± 0.08 | 0.63 ± 0.53 |  |  |  |  |  |  |  |  |  |
|  |  | 2015 | 0.34 ± 0.14 | 0.72 ± 0.71 |  |  |  |  |  |  |  |  |  |
|  |  | 2016 | 0.22 ± 0.06 | 0.42 ± 0.39 |  |  |  |  |  |  |  |  |  |
|  |  | 2017 | 0.33 ± 0.13 | 0.63 ± 0.58 |  |  |  |  |  |  |  |  |  |

^a^ PIFL= *Pinus flexilis* (limber pine); PILO = *Pinus longaeva* (bristlecone pine).

**Supplementary Table S2:** Regression parameters of the linearized equation [Log (*y*) = *a* × Log (*x*) – *b*] for weekly basal area increment (BAI, cm^2^ wk^-1^) and transpiration (*T*, m^3^ wk^-1^) vs. weekly mean vapor pressure deficit (VPD, kPa) and soil moisture at 20 cm depth (SM_20_, %) during the early and late growing seasons (Figure 4). Differences in slopes (parameter *a*) between the early and late growing seasons were tested using ANCOVA.

|  | Environmental  variables | Parameters | Early season | Late season | *F*-test | *P*-value |
| --- | --- | --- | --- | --- | --- | --- |
| BAI | VPD | *a* | –0.29 | –1.86 |  |  |
|  |  | *b* | 0.08 | 0.96 |  |  |
|  |  | *R^2^* | 0.01 | 0.57 |  |  |
|  |  | *P* | n.s. | < 0.001 |  |  |
|  | SM_20_ | *a* | 0.51 | 2.30 |  |  |
|  |  | *b* | 0.59 | 2.75 |  |  |
|  |  | *R^2^* | 0.03 | 0.40 |  |  |
|  |  | *P* | n.s. | < 0.001 |  |  |
| *T* | VPD | *a* | 0.25 | –0.12 |  |  |
|  |  | *b* | 0.98 | 1.23 |  |  |
|  |  | *R^2^* | 0.03 | 0.01 |  |  |
|  |  | *P* | n.s. | n.s. |  |  |
|  | SM_20_ | *a* | 0.42 | 0.55 | 0.19 | 0.661 |
|  |  | *b* | 1.41 | 1.70 |  |  |
|  |  | *R^2^* | 0.06 | 0.09 |  |  |
|  |  | *P* | < 0.05 | < 0.05 |  |  |

**Supplementary Figure S1.** Time series of 3-month standardized precipitation evapotranspiration index (SPEI; data obtained from https://spei.csic.es/index.html) for the 0.5^o^ × 0.5^o^ gridded cell (38.75N, 114.25W) that includes the study area. Blue (positive values) and red (negative values) areas denote wet and dry periods, respectively.

**Supplementary Figure S2.** Relative changes in water use efficiency (*Δ*WUE) calculated for each calendar week as the deviation of weekly WUE from the corresponding 5-year average. Limber pine (PIFL) and bristlecone pine (PILO) values are shown against a gray background when REW < 0.4 (see Figure 1).
